# No-Reference Image Quality Assessment with Global Statistical Features

**DOI:** 10.3390/jimaging7020029

**Published:** 2021-02-05

**Authors:** Domonkos Varga

**Affiliations:** Department of Networked Systems and Services, Budapest University of Technology and Economics, 1111 Budapest, Hungary; dvarga@edu.bme.hu

**Keywords:** no-reference image quality assessment, Benford’s law, quality-aware features, image statistics

## Abstract

The perceptual quality of digital images is often deteriorated during storage, compression, and transmission. The most reliable way of assessing image quality is to ask people to provide their opinions on a number of test images. However, this is an expensive and time-consuming process which cannot be applied in real-time systems. In this study, a novel no-reference image quality assessment method is proposed. The introduced method uses a set of novel quality-aware features which globally characterizes the statistics of a given test image, such as extended local fractal dimension distribution feature, extended first digit distribution features using different domains, Bilaplacian features, image moments, and a wide variety of perceptual features. Experimental results are demonstrated on five publicly available benchmark image quality assessment databases: CSIQ, MDID, KADID-10k, LIVE In the Wild, and KonIQ-10k.

## 1. Introduction

As digital media takes a more central part in our daily lives and work, research on image and video quality assessment becomes more and more important. In many cases, the visual quality has to be optimized for content, like movies and sport games. In these cases, automatic assessment methods should take the actual image or video content into account to give the viewer the best experience. In medical imaging, a poor image quality may mean a misdiagnosis.

There are two ways of measuring image quality [1]. The obvious way is to ask people to give their opinions on a number of test images which is called subjective quality assessment. However, such a procedure can be very time-consuming and expensive to set up the experimental environment. That is why, objective image quality assessment has become a hot research topic because it deals with mathematical models and algorithms that are able to assess perceptual quality of digital images automatically. In the literature, objective image quality assessment algorithms are grouped according to the availability of the reference, pristine image. Specifically, full-reference image quality assessment (FR-IQA) algorithms possess full information for both the distorted image and the reference image, while no-reference image quality assessment (NR-IQA) methods predict perceptual quality exclusively based on the distorted image. Reduced-reference image quality assessment (RR-IQA) represents a middle course because it possess partial information about the reference image and full information about the distorted image.

### 1.1. Related Work

NR-IQA has gained a lot of attention in the recent decades. Although, the reference image is not available for NR-IQA algorithms, they can make assumptions about the distortions present in a given input image. Hence, they can be divided into distortion-specific and general-purpose groups. As the name indicates, distortion-specific methods assume the presence of certain distortions, such as JPEG [2] or JPEG2000 [3] compression noise. In contrast, general-purpose algorithms do not restrict themselves to specific distortions. General-purpose methods can be further divided into opinion-unaware [4,5] and opinion-aware [6] classes. Opinion-unaware ones do not require subjective quality scores in the training process, while opinion-aware algorithms usually rely on different regression frameworks trained on subjective scores.

Models based on natural scene statistics (NSS) have been very popular in opinion-aware NR-IQA. The main idea is that pristine (distortion-free) images obey certain statistical regularities and distorted images’ statistics deviate significantly from these regularities. As a consequence, these models contain three distinct stages: (1) feature extraction, (2) NSS modeling, and (3) regression. Hence, the main differences between NSS-based algorithms are connected to the above-mentioned three steps. For instance, blind image quality index (BIQI) [7] extracts features in the wavelet domain over three scales and three orientations. Moreover, generalized Gaussian distribution is fitted to the sub-band coefficients and the fitting parameters are utilized as quality-aware features. Finally, a trained support vector regressor (SVR) is applied to map features onto perceptual quality scores. In contrast, blind image integrity notator using DCT statistics (BLIINDS) [8] utilizes the statistics of local DCT coefficients. On the other hand, the mapping from features to quality scores is carried out by probabilistic prediction algorithms. In contrast, Liu et al. [9] utilized the orientation information from curvelet transform to determine correlation between scale and orientation energy distributions. Similarly to [7], an SVR is used to map the feature vectors onto quality scores. Gu et al. [10] combined NSS-based features with the free energy principle. He et al. [11] integrated NSS-based features and sparse representation. Mittal et al. [6] extracted NSS-based features from the spatial domain. Namely, mean subtracted contrast normalized (MSCN) coefficients were first determined from the raw pixel data. Subsequently, a generalized Gaussian distribution was fitted to MSCN coefficients. Moreover, an asymmetric generalized Gaussian distribution was also fitted to the products of neighboring MSCN coefficients. Similarly to BIQI [7], the fitting parameters were considered as quality-aware features and mapped to perceptual quality scores with a SVR. In [12], NSS features from multiple domains were combined. In contrast, Jenadeleh and Moghaddam [13] estimated the parameters of NSS features by a Wakeby distribution model.

Another line of papers extracts directly quality-aware statistical features from images and maps them to quality scores. Zhang et al. [14] generated quality-aware features from the joint generalized local binary pattern statistics. In contrast, Li et al. [15] proposed a gradient weighted histogram of local binary patterns for quality aware features. In [16], a set of quality aware statistical features (first digit distribution in the gradient magnitude and wavelet domain, color statistics) were combined with powerful perceptual features (colorfulness, global contrast factor, entropy, etc.) to train an Gaussian process regression (GPR) algorithm for quality prediction.

Recently, convolutional neural networks have become a prominent technology in the field of image processing. The deployment of CNNs in NR-IQA is gaining a lot of attention due to their representational power. Usually, CNNs consist of four types of components, such as convolutional, activation, pooling, and fully-connected layers stacked on each other. On the other hand, features extracted from CNNs trained on huge databases, such as ImageNet [17], have shown excellent representational power in many image processing tasks [18,19,20]. First, Kang et al. [21] proposed a CNN-based solution for NR-IQA. Specifically, the authors trained a CNN regression framework on 32×32 non-overlapping image patches. The perceptual quality of the overall input image was determined by pooling the patches’ quality scores. Later, the proposed architecture was developed further by Kang et al. [22] to simultaneously estimate perceptual quality and image distortion types. Similarly, Kim et al. [23] proposed a regression CNN framework, but FR-IQA behavior was first imitated by generating a local quality map. Namely, the patches were first regressed onto quality scores obtained by a traditional FR-IQA metric. In contrast, Bianco et al. [24] applied fine-tuned AlexNet [25] to extract deep features from 227×227-sized image patches. Zeng et al. [26] developed the approach of [24] further. Namely, they extracted features with the help of a ResNet [27] architecture and elaborated a probabilistic representation of distorted images. In [28], an Inception-V3 [29] network was utilized as feature extracted and it was pointed out that considering the features of multiple layers is able to improve the performance of perceptual quality prediction. In contrast, Liu et al. [30] trained a Siamese CNN to rank images in terms of perceptual quality. Subsequently, the trained Siamese CNN was used to transfer knowledge into a traditional CNN. Lin and Wang [31] proposed a quality-aware generative network for reference image generation. To this end, a quality-aware loss function was also proposed. Moreover, the knowledge about the discrepancy between real and generated reference images was incorporated into a regression CNN which estimated the perceptual quality of distorted images.

Another line of NR-IQA algorithms focuses on combining the results of existing methods to improve prediction performance [32,33]. For instance, Ieremeiev et al. [34] trained a neural network on the results of eleven different NR-IQA algorithms to boost performance.

### 1.2. Contributions

In this study, an NR-IQA method is presented which relies on a novel feature vector containing a set of quality-aware features that globally characterizes the statistics of a given input image to be assessed. Specifically, the proposed feature vector partially improves further our previous work [16]. A set of shape descriptors is proposed to the local fractal dimension distribution and first digit distribution feature vectors to capture better image distortions. Moreover, we point out that besides the wavelet coefficients [16], discrete cosine transform coefficients and singular values of an image are also suitable to derive first digit distribution features based on Benford’s law. Motivated by the model of extended classical receptive field (ECRF), Bilaplacian quality-aware features are also incorporated into the introduced model. Unlike previous methods, the degradation of image edges are quantified by image moments. Experimental results and performance comparison to the state-of-the-art are presented on five publicly available IQA benchmark databases: CSIQ, MDID, KADID-10k, LIVE In the Wild, and KonIQ-10k.

### 1.3. Structure

The rest of this study is organized as follows. Section 2 describes our proposed method for NR-IQA. Next, Section 3 shows experimental results and analysis including the description of the applied IQA benchmark databases and evaluation protocol, a parameter study, and a comparison to other state-of-the-art algorithms. Finally, a conclusion is drawn in Section 4.

## 2. Proposed Method

**Figure 1:**
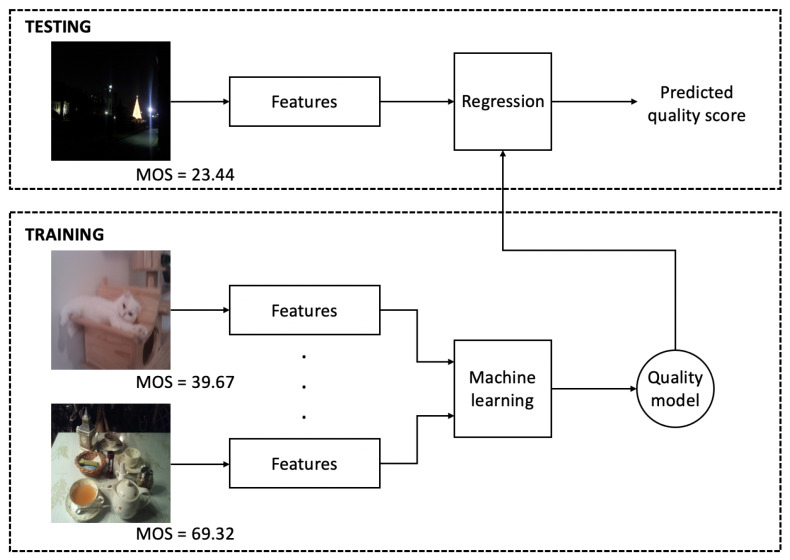
Block diagram of the proposed method.

The general overview of the proposed NR-IQA method is shown in Figure 1. As the workflow indicates, a set of feature vectors is extracted from the training images to train a machine learning model which is applied in the testing phase for mapping feature vectors into perceptual quality scores. In Section 3, a detailed parameter study is presented to find the most suitable regression using five IQA benchmark databases.

As pointed out by Ghadiyaram and Bovik [35], a various set of features is necessary to accurately predict artificially and authentically distorted digital images’ perceptual quality. In this study, a novel set of quality-aware features is proposed that characterizes an image by taking into account its global statistics. The introduced method relies on a 132-dimensional feature vector including extended local fractal dimension distribution feature vector, extended first digit distribution (FDD) feature vectors, Bilaplacian features, image moments, histogram variances of relative gradient orientation (RO), gradient magnitude (RM), relative gradient magnitude (GM) maps, and perceptual features (colorfulness, sharpness, dark channel feature, contrast). The used features are summarized in Table 1 where quality-aware features proposed by this study are typed in bold.

### 2.1. Extended Local Fractal Dimension Distribution Feature Vector

**Figure 2:**
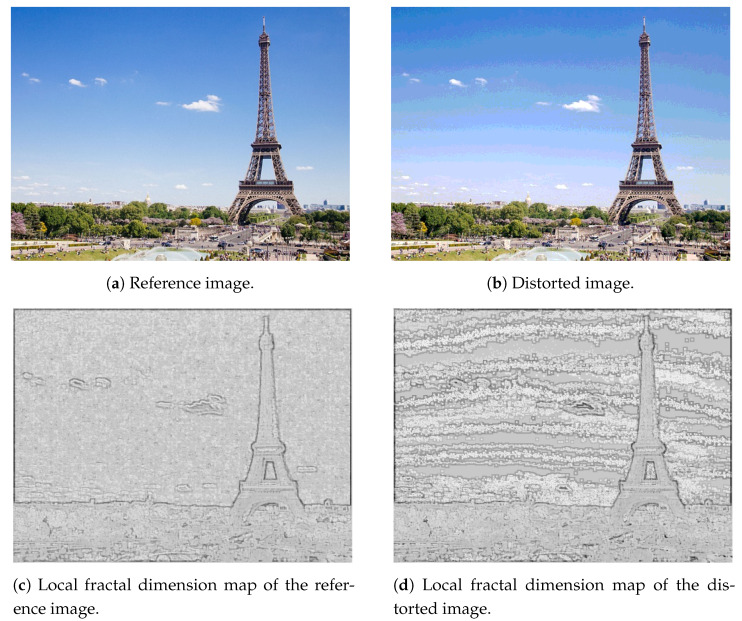
Illustration of local fractal dimension maps.

In [41], Pentland demonstrated that natural scenes, such as mountains, trees, clouds, etc., can be described by fractal surfaces because fractals look like as natural surfaces. Various image distortions often change the local regularities of digital images’ texture. Thus, distortions change the local fractal dimension distribution of a given test image. Consequently, the histogram of local fractal dimension distributions are quality-aware features [16]. As in our previous study [16], the local fractal dimension map of an image is created by considering each pixel in the original image as a center of a 7-by-7 rectangular neighborhood and the fractal dimension is calculated from this neighborhood. The box-counting method is applied to determine the fractal dimension of an image patch because it is able to represent complexity and easy to implement [42,43]. Similarly to our previous work [16], a 10-bin normalized histogram was calculated considering the values between −2 and 3 from the local fractal dimension map. Figure 2 depicts the local fractal dimension maps of a reference-distorted image pair. It can be observed that distortions in texture appear very strongly in the local fractal dimension.

Although the normalized histogram of local fractal dimension distribution is able to describe the irregularities of natural scene, the following statics are attached to the normalized histogram to construct an effective feature vector: skewness, kurtosis, entropy, median, spread, and standard deviation. The skewness is determined as

(1) $$s\left(v\right)=\frac{\bar{{\left(v-\bar{v}\right)}^{3}}}{std(v)},$$

where $\bar{v}$ stands for the mean of *v* and std(v) is the standard deviation of *v*. The kurtosis is obtained as

(2) $$k\left(v\right)=\frac{\bar{{\left(v-\bar{v}\right)}^{4}}}{std{\left(v\right)}^{4}}-3.$$

The entropy is obtained as

(3) $$e\left(v\right)=-\sum_{i}p_{i}\left(v\right)\log_{2}p_{i}\left(v\right),$$

where $p_{i}\left(v\right)$ stands for the histogram count of *v*.

### 2.2. Extended First Digit Distribution Feature Vectors

Benford’s distribution concerns the leading digit (the first non-zero digit, range: 1–9) of values in a data set. Frank Benford published an article entitled “The law of anomalous numbers” in 1938 [44] where he analyzed the leading digit values from diverse sources, such as populations of counties, length of rivers, or death rates. Benford conjectured that the distribution of the leading digit x=1,2,…,9 has probability mass function

(4) $$f\left(x\right)=\log_{10}\left(1+\frac{1}{x}\right).$$

Those data sets, that follows the particular pattern defined by Equation (4) for their leading digits, are said to satisfy Benford’s law. It was pointed out by Pérez-González et al. [45] that the luminance values of digital images do not satisfy Benford’s law. However, the discrete cosine transform (DCT) coefficients of a digital image produces a good match with Benford’s law [45].

In our previous work [16], we utilized the wavelet domain to obtain first digit distribution (FDD) feature vectors, since we pointed out that the FDD in the wavelet transform domain matches very well with the Benford’s law prediction in case of distortion-free, pristine images. On the other hand, various image distortions result in a significant deviation from the prediction of the Benford’s law in FDD. However, the discrete cosine transform (DCT) coefficients’ and singular values’ FDD shows similar properties to those of wavelet domain. In this study, normalized FDD feature vectors are extracted from the DCT coefficients [45] and the singular values, besides the wavelet transform domain. Moreover, the FDD distribution feature vectors are augmented by statistics as in the previous subsection, such as symmetric Kullback–Leibler divergence between the actual FDD and Benford’s law prediction, skewness, kurtosis, entropy, median, spread, and standard deviation. As already mentioned, symmetric Kullback–Leibler (sKL) divergence is determined between the actual FDD (denoted by P(x),x=1,2,…,9) and Benford’s distribution (denoted by B(x),x=1,2,…,9):

(5) $$sKL\left(P\left(x\right),B\left(x\right)\right)=\frac{1}{2}KL\left(P\left(x\right),B\left(x\right)\right)+\frac{1}{2}KL\left(B\left(x\right),P\left(x\right)\right),$$

where the Kullback–Leibler (KL) divergence is given as:

(6) $$KL\left(P\left(x\right),B\left(x\right)\right)=\sum_{x=1}^{9}P\left(x\right)\log_{2}\frac{P(x)}{B(x)}.$$

In addition to sKL, skewness, kurtosis, entropy, median, spread, and standard deviation were also attached to the normalized FDD to obtain the extended FDD feature vector. As a result, an extended FDD feature vector has a length of 17. Moreover, extended FDD feature vectors are extracted from the horizontal, vertical, and diagonal wavelet coefficients, DCT coefficients, and singular values.

Table 2 illustrates the average FDD of singular values in the KADID-10k [46] database with respect to the five different distortion levels found in this database. It can be observed that the sKL between the actual FDD and the Benford’s distribution is roughly proportional with the level of distortion. Furthermore, the relative frequency of ones and twos are also roughly proportional with the level of image distortion. That is why, the FDDs in different domains were chosen as quality-aware descriptors and were extended with sKL and histogram shape descriptors, such as skewness, kurtosis, entropy, median, spread, and standard deviation.

### 2.3. Bilaplacian Features

Gerhard et al. [47] pointed out that the human visual system (HVS) is adapted to the statistical regularities in images. Moreover, Marr [48] emphasized the importance of studying zero-crossings at multiple scales to interpret the intensity changes found in the image. At the same time, the extended classical receptive field (ECRF) of retinal ganglion cells can be modeled as a combination of three zero-mean Gaussians at three different scales [49]. These are equivalent to a Bilaplacian of the Gaussian filter [49,50]. On the other hand, Gaussian filtering introduces an undesirable distortion in IQA. In our method, YCbCr color space is applied, since it is suggested by ITU-R BT.601 for video broadcasting to obtain Bilaplacian features. The direct conversion from RGB color space to YCbCr is the following:

(7) $$\left(\begin{array}{c} Y\\ Cb\\ Cr \end{array}\right)=\left(\begin{array}{ccc} 0.2568 & 0.5041 & 0.0979\\ -0.1482 & -0.2910 & 0.4392\\ 0.4392 & -0.3678 & -0.0714 \end{array}\right)\left(\begin{array}{c} R\\ G\\ B \end{array}\right),$$

where *R*, *G*, and *B* denote the red, green, and blue color channels, respectively.

Generally, the Laplacian filters are approximated by convolution kernels whose sum are zero [51]. In this paper, the following popular kernels are utilized:

(8) $$L_{1}=\left(\begin{array}{ccc} 0 & 1 & 0\\ 1 & -4 & 1\\ 0 & 1 & 0 \end{array}\right),L_{2}=\left(\begin{array}{ccc} 1 & -2 & 1\\ -2 & 4 & -2\\ 1 & -2 & 1 \end{array}\right),L_{3}=\left(\begin{array}{ccc} 1 & 0 & 1\\ 0 & -4 & 0\\ 1 & 0 & 1 \end{array}\right),L_{4}=\left(\begin{array}{ccc} -2 & 1 & -2\\ 1 & 4 & 1\\ -2 & 1 & -2 \end{array}\right),$$

(9) $$L_{5}=\left(\begin{array}{ccc} -1 & -1 & -1\\ -1 & 8 & -1\\ -1 & -1 & -1 \end{array}\right).$$

An image can be converted to the Bilaplacian domain by convolving it with two Laplacian kernels, formally can be written as:

(10) $$L_{ij}^{2}*I=L_{i}*L_{j}*I,$$

where ∗ stands for the operation of convolution. In our study, $L_{11}^{2}$, $L_{22}^{2}$, $L_{33}^{2}$, $L_{44}^{2}$, $L_{55}^{2}$, $L_{13}^{2}$, and $L_{24}^{2}$ masks are considered. As already mentioned, the channels of YCbCr color space are used to obtain the Bilaplacian features. This means that *Y*, Cb, and Cr channels are convolved with the Bilaplacian masks independently from each other. As a consequence, seven Bilaplacian maps can be obtained for each color channel. Subsequently, the histogram variance of each channels is taken. The histogram variance is defined as

(11) $$hvar\left(v\right)=\sum_{v}{\left(h\left(v\right)-\bar{v}\right)}^{2},$$

where h(v) stands for *v*’s normalized histogram to unit sum.

**Figure 3:**
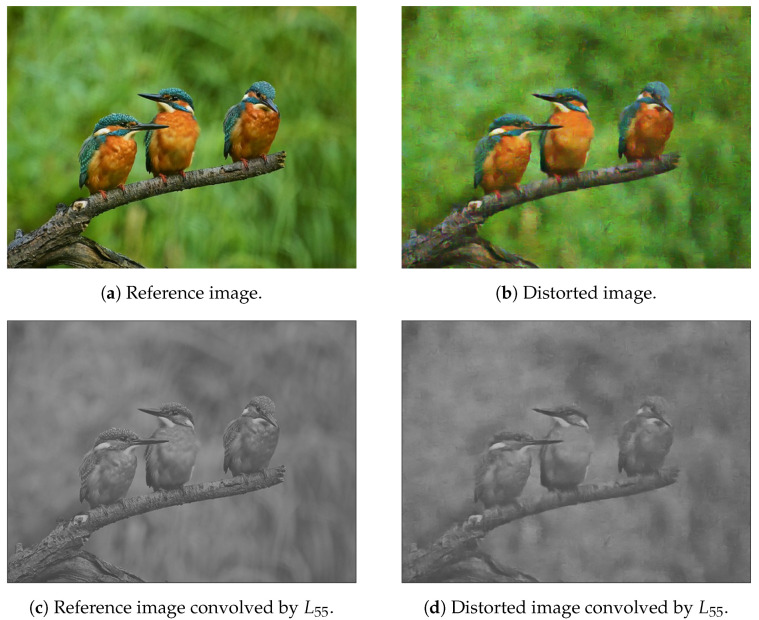

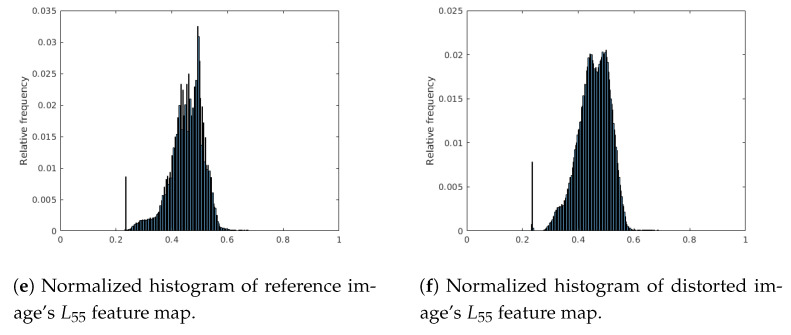
Illustration of Bilaplacian features. The first row contains a reference-distorted image pair. The second row consists of the Bilaplacian feature maps obtained by the $L_{55}$ filter. The third row contains the normalized histograms of the Bilaplacian feature maps.

Figure 3 illustrates a reference, distortion-free image and its artificially distorted counterpart from the KADID-10k [46] database. It can be seen that even a moderate amount of noise can significantly distort the normalized histogram of Bilaplacian feature maps. That is why the histogram variances of the Bilaplacian feature maps were applied as quality-aware features.

### 2.4. Image Moments

A number of IQA metrics have utilized that the structural distortions of digital images correlate well with the degradation of edges [52,53]. In this paper, we propose to use the global, binary Sobel edge map of a digital image and determine the eight central moments ((0,2),(0,3),(1,1),(2,1),(1,2),(2,0),(2,1),(3,0)) which are used as quality-aware features.

First, the Sobel operator computes an approximation of the gradient of an image. If I is considered as the source image, $G_{x}$ and $G_{y}$ are determined as:

(12) $$G_{x}=\left(\begin{array}{ccc} +1 & 0 & -1\\ +2 & 0 & -2\\ +1 & 0 & -1 \end{array}\right)*I,$$

(13) $$G_{y}=\left(\begin{array}{ccc} +1 & +2 & +1\\ 0 & 0 & 0\\ -1 & -2 & -1 \end{array}\right)*I,$$

where ∗ stands for the convolution operator, $G_{x}$ and $G_{y}$ are the horizontal and vertical derivative approximations, respectively. The gradient magnitude approximations can be obtained:

(14) $$G=\sqrt{G_{x}^{2}+G_{y}^{2}}.$$

**Figure 4:**
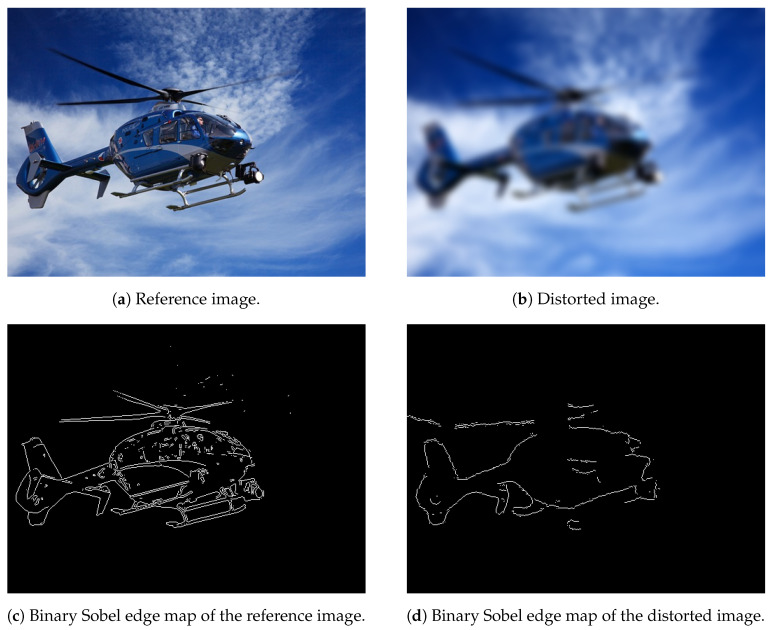
Structural distortions correlate well with the degradation. Central moments are applied as quality-aware features to quantify edge degradation.

The binary Sobel edge map is determined by thresholding G using the quadruple of G’s mean as cutoff threshold. Finally, edge thinning is applied to remove spurious points from the edge map [54]. The central moments of the digital image I(x,y) are defined as

(15) $$\mu_{pq}=\sum_{x}\sum_{y}{\left(x-\bar{x}\right)}^{p}{\left(y-\bar{y}\right)}^{q}I\left(x,y\right),$$

where $\bar{x}$ and $\bar{y}$ are the coordinates of the binary image’s centroid. By definition, the centroid of a binary image is the arithmetic mean of all (x,y) coordinates. It can be shown that central moments are translational invariant [55] (Figure 4).

### 2.5. Gradient Features

Image gradient magnitude and orientation features have become very popular both in FR-IQA and NR-IQA since they are strong predictive factors of perceptual image quality [36]. In this study, the histogram variances of gradient magnitude (GM), relative gradient orientation (RO), and relative gradient magnitude (RM) are incorporated into our model to quantify the changes in gradient [36].

### 2.6. Perceptual Features

The following perceptual features are adopted in our model, since they are coherent with the HVS’s quality perception. Specifically, colorfulness [37], sharpness [38], dark channel feature [39], and contrast [40] were applied in our study.

Yendrikhovskij et al. [56] demonstrated that colorfulness plays an important role in human perceptual quality judgments, since humans like better more colorful images. In this study, the metric of Hasler and Suesstrunk [37] was adopted:

(16) $$CF=\sqrt{\sigma_{rg}^{2}+\sigma_{yb}^{2}}+\frac{3}{10}\sqrt{\mu_{rg}^{2}+\mu_{yb}^{2}},$$

where rg=R−G and $yb=\frac{1}{2}\left(R+G\right)-B$. Furthermore, *R*, *G*, and *B* stand for the red, green, and blue channels, respectively. Variables σ and μ denote the standard deviation and mean of the matrices given in the subscripts, respectively.

Image sharpness determines the amount of detail that is realized in the image. Sharpness can be observed most clearly on image edges and for that reason it is widely considered as an image quality factor. In this study, the metric of Bahrami and Kot [38]—maximum local variation (MLV)—was adopted to characterize the sharpness of an image because its low computational costs.

First, Tang et al. [57] proposed dark channel features for photo quality assessment. Dark channel features were designed originally for single image haze removal [39]. An image’s (I) dark channel $\left(I_{dark}\right)$ is defined as:

(17) $$I_{dark}\left(i\right)=\min_{c\in \{R,G,B\}}\left(\min_{i^{\prime }\in \Omega \left(i\right)}I_{c}\left(i^{\prime }\right)\right),$$

where $I_{c}$ is a color channel of *I*(c∈{R,G,B}) and Ω(i) is a neighborhood of pixel *i*. In our implementation, Ω(i) is a rectangular 15×15-sized patch. The dark channel feature of image *I* is defined as:

(18) $$DCF=\frac{1}{\left||S|\right|}\sum_{i\in S}\frac{I_{dark}\left(i\right)}{\sum_{c\in \{R,G,B\}}I_{c}\left(i\right)},$$

where *S* denotes the area of the input image.

There are many definitions of image contrast in the literature. The easiest way to explain contrast is the difference between the brightest and darkest pixel values. Therefore, the HVS’s capability to recognize and separate objects on an image heavily depends on image contrast. Consequently, contrast is an image quality factor. In this study, Matkovic et al.’s [40] global contrast factor (GCF) model was adopted which is defined as follows:

(19) $$GCF=\sum_{i=1}^{9}w_{i}C_{i},$$

where $w_{i}=\left(-0.406385\;\cdot \;\frac{i}{9}+0.334573\right)\;\cdot \;\frac{i}{9}+0.0877526$, i∈{1,2,…,9}. Moreover, $C_{i}$s are defined as

(20) $$C_{i}=\frac{1}{w\;\cdot \;h}\sum_{i=1}^{w\;\cdot \;h}l_{C_{i}},$$

where *w* and *h* stand for the width and height of the input image, respectively, and

(21) $$l_{C_{i}}=\frac{|L_{i}-L_{i-1}\left|+\right|L_{i}-L_{i+1}\left|+\right|L_{i}-L_{i-w}\left|+\right|L_{i}+L_{i+w}|}{4},$$

where the *L*s denote the pixel values after gamma correction (γ=2.2) and assuming that the image is reshaped into a row-wise one dimensional array.

Table 3 illustrates the average values of the applied perceptual features(CF, sharpness, DCF, and GCF) in the KADID-10k [46] database with respect to the five different distortion levels. It can be observed that the applied four perceptual features strongly correlate with the distortion levels.

## 3. Experimental Results

In this section, our experimental results are presented. Section 3.1 gives a brief overview about the used publicly available IQA benchmark databases. Next, Section 3.2 describes the used experimental setup and evaluation metrics. Section 3.3 contains a parameter study in which our design choices are reasoned. Subsequently, Section 3.4 consists of a performance comparison to other state-of-the-art NR-IQA algorithms using publicly available IQA benchmark databases. Finally, Section 3.5 and Section 3.6 contain detailed results with respect to distortion types and levels.

### 3.1. Databases

Five publicly available benchmark IQA databases are used in this study to demonstrate and validate the results of the proposed method including CSIQ [58], KADID-10k [46], MDID [59], LIVE In the Wild [35], and KonIQ-10k [60] datasets.

CSIQ [58] has 30 reference images, each one distorted by one of six predefined distortion types at four or five different distortion levels. MDID [59] contains 20 reference images and 1600 distorted images derived from the reference images using multiple distortions of random types and distortion levels. Moreover, the authors [59] proposed a novel subjective rating method, called pair comparison sorting, to obtain more accurate data. KADID-10k [46] consists of 10,125 distorted images derived from 81 pristine (distortion free), reference images using 25 different distortion types at 5 different distortion levels. Moreover, each image is associated with a differential MOS value in the range of [1, 5]. In contrast, LIVE In the Wild [35] database contains images captured by mobile camera devices so the images are affected by an intricate mixture of different distortion types. In total, it contains 1162 authentically distorted images which were evaluated by 8100 human observers. Similarly, KonIQ-10k [60] database consists of digital images with authentic distortions. Specifically, 10,073 images were sampled from the YFCC100m [61] database using seven quality indicators, one content indicator, and machine tags. Moreover, 120 quality ratings were collected for all images using crowd sourcing platforms.

Table 4 presents a comparison of the applied IQA benchmark databases with respect to their main characteristics.

### 3.2. Experimental Setup and Evaluation Metrics

To evaluate our model and other state-of-the-art algorithms, databases containing artificial distortions (CSIQ [58], MDID, and KADID-10k [46]) are divided into a training set and a test with respect to the pristine, reference images to avoid any semantic content overlapping between these two sets. Databases with authentic distortions (LIVE In the Wild) are simply divided into a training and a test set. Moreover, approximately 80% of images are in the training set and the remaining 20% are in the test. In this study, two widely applied correlation criteria are employed including Pearson’s linear correlation coefficient (PLCC) and Spearman’s rank order correlation coefficient (SROCC). For both PLCC and SROCC, a higher value indicates a better performance of the examined NR-IQA algorithm. Furthermore, we report average PLCC and SROCC values which were measured over 100 random train–test splits.

### 3.3. Parameter Study

**Figure 5:**
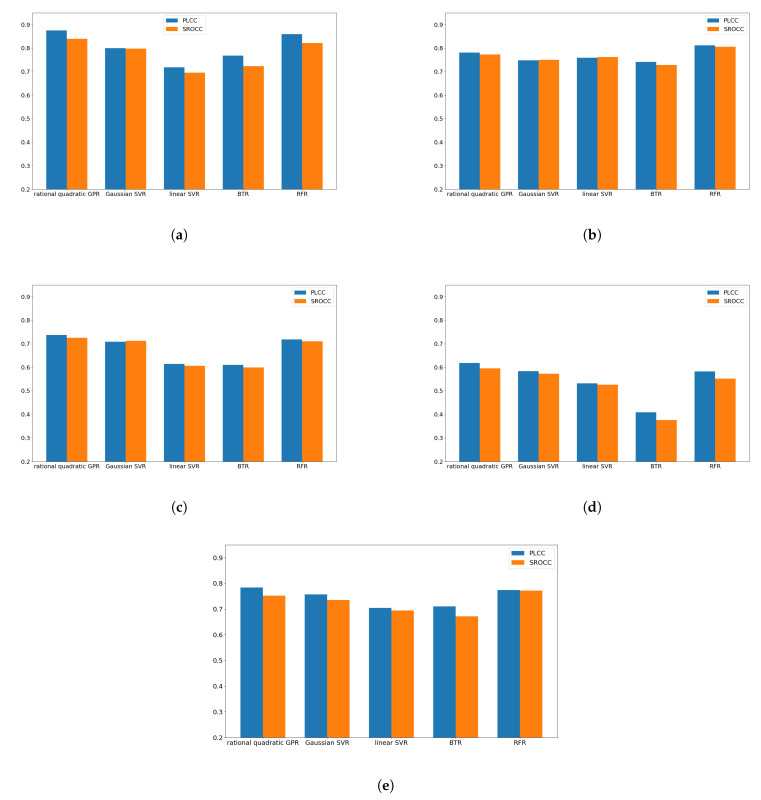
Performance comparison of rational quadratic Gaussian process regressor (GPR), Gaussian support vector regressor (SVR), linear SVR, binary tree regression (BTR), and random forest regression (RFR) techniques. Average Pearson’s linear correlation coefficient (PLCC) and Spearman’s rank order correlation coefficient (SROCC) values measured 100 random train–test splits are plotted. (**a**) CSIQ [58]. (**b**) MDID [59]. (**c**) KADID-10k [46]. (**d**) LIVE In the Wild [35]. (**e**) KonIQ-10k [60].

In this subsection, a parameter study is carried out to find an optimal regression technique for the proposed quality-aware global statistical features. Specifically, we made experiments with five different regression algorithms, such as rational quadratic Gaussian process regressor (GPR) [62], Gaussian support vector regressor (SVR) [63], linear SVR [63], binary tree regression (BTR) [64], and random forest regression (RFR) [65]. The results are summarized in Figure 5. It can be seen that rational quadratic GPR provides the best performance on CSIQ [58], KADID-10k [46], and LIVE In the Wild [35]. On MDID [59], RFR provides the best results, while rational quadratic GPR is the second best. On KonIQ-10k [60], rational quadratic GPR and RFR give similar results. As a consequence, rational quadratic GPR was chosen in our method. Moreover, this architecture is codenamed *GSF-IQA* in the following subsections and compared to the state-of-the-art.

### 3.4. Comparison to the State-of-the-Art

To compare our *GSF-IQA* method to the state-of-the-art, several NR-IQA methods were collected whose original source codes are available online, including BLIINDS-II [66], BMPRI [67], BRISQUE [6], CurveletQA [9], DIIVINE [68], ENIQA [69], GRAD-LOG-CP [70], NBIQA [71], PIQE [4], OG-IQA [36], SPF-IQA [16], and SSEQ [72].

As already mentioned, five benchmark IQA databases are used in this study: CSIQ [58], MDID [59], KADID-10k [46], LIVE In the Wild [35], and KonIQ-10k [60]. The measured results of the proposed method and other state-of-the-art algorithms on artificial distortions (CSIQ [58], MDID [59], and KADID-10K [46]) can be seen in Table 5, while those on authentic distortions (LIVE In the Wild [35] and KonIQ-10k [60]) are summarized in Table 6. In addition to this, Table 7 presents the results of the one-sided *t*-test which was applied to give evidence for the statistical significance of *GSF-IQA*’s results on the used IQA benchmark databases. In this table, each record is encoded by two symbols. Namely, ‘1’ means that the proposed *GSF-IQA* method is statistically significantly better than the NR-IQA method in the row on the IQA benchmark database in the column. The ‘-’ symbol is adopted when there is no significant difference between GSF-IQA and another NR-IQA method. Table 8 illustrates the weighted and direct average of PLCC and SROCC values found in Table 5 and Table 6.

From the results presented in Table 5, Table 6, Table 7 and Table 8, it can be seen that the proposed *GSF-IQA* provides the best results on four out of five IQA benchmark databases. Moreover, it gives the second best PLCC and SROCC values on LIVE In the Wild [35]. From the significance tests, it can be observed that the improvement is statistically significant on all databases containing artificial distortions. On the other hand, the difference between the best and the second best performing methods on LIVE In the Wild [35] and KonIQ-10k [60] is statistically not significant. It can be also observed from Table 8 that the proposed *GSF-IQA* method is able to outperform other state-of-the-art algorithms in terms of direct and weighted average PLCC and SROCC values. Specifically, *GSF-IQA* outperforms the second best method by approximately 0.02 both in terms of direct and weighted average PLCC and SROCC values.

**Figure 6:**
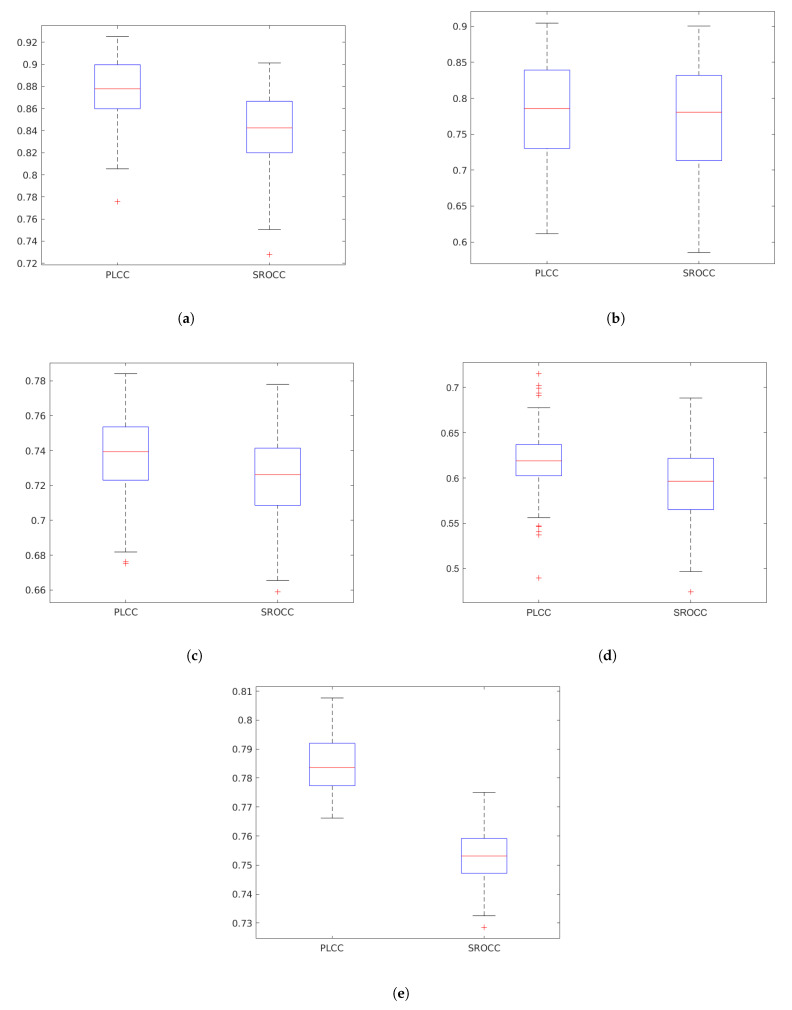
Box plots of the PLCC and SROCC values produced by GSF-IQA on five IQA benchmark databases (CSIQ [58], MDID [59], KADID-10k [46], LIVE In the Wild [35], and KonIQ-10k [60]). Measured over 100 random train–test splits. (**a**) CSIQ [58]. (**b**) MDID [59]. (**c**) KADID-10k [46]. (**d**) LIVE In the Wild [35]. (**e**) KonIQ-10k [60].

Figure 6 depicts the boxplots of PLCC and SROCC values produced by *GSF-IQA* on each applied IQA benchmark database. On every box, the red central mark stands for the median value, and the blue bottom and top edges of the box denote the 25th and 75th percentiles, respectively. In addition, the whiskers indicate the most extreme values which are not considered as outliers. The outliers are depicted by ’+’.

### 3.5. Performance over Different Distortion Types

In this subsection, we examine the performance of the state-of-the-art NR-IQA methods over different distortion types. Specifically, we report on average SROCC values measured over the different distortion types of KADID-10k database [46]. As already mentioned, this database consists of images with 25 different distortion types, such as Gaussian blur (GB), lens blur (LB), motion blur (MB), color diffusion (CD), color shift (CS), color quantization (CQ), color saturation 1 (CSA1), color saturation 2 (CSA2), JPEG2000 compression noise (JP2K), JPEG compression noise (JPEG), white noise (WN), white noise in color component (WNCC), impulse noise (IN), multiplicative noise (MN), denoise, brighten, darken, mean shift (MS), jitter, non-eccentricity patch (NEP), pixelate, quantization, color block (CB), high sharpen (HS), and contrast change (CC). The results are summarized in Table 9. It can be seen that the proposed *GSF-IQA* algorithm is able to provide the best results on 12 out of 25 distortion types.

### 3.6. Performance over Different Distortion Levels

In this subsection, we examine the performance of the state-of-the-art NR-IQA methods over different distortion levels. Specifically, we report on average SROCC values measured over the different distortion levels of the KADID-10k database [46]. The results are summarized in Table 10. As one can see from the results, the proposed *GSF-IQA* algorithm is able to outperform all the other state-of-the-art methods on all distortion levels.

## 4. Conclusions

In this paper, we proposed a novel NR-IQA algorithm based on a set of novel quality-aware features which globally characterizes the statistics of an image. First, we utilized that various image distortions change the local regularities of the texture. Thus, an extended local fractal dimension feature was proposed to quantify the texture’s degradation. Second, we demonstrated that first digit distributions of wavelet coefficients, DCT coefficients, and singular values can be used as quality-aware features and proposed extended first digit distribution feature vectors. This model was improved by Bilaplacian features which was inspired by the extended classical receptive field model of retinal ganglion cells. To quantify the degradation of edges, image moments were incorporated into the model. The proposed algorithm was tested on five publicly available benchmark databases including CSIQ, MDID, KADID-10k, LIVE In the Wild, and KonIQ-10k. It was demonstrated that our proposal is able to outperform other state-of-the-art methods both on artificial and authentic distortions. There are two main directions of future research. Beyond feature concatenation, it is worth to study the selection process of relevant attributes provided by different sources. Moreover, the incorporation of local statistical features provided by local feature descriptors may improve the performance, since some distortion types do not uniformly distribute in the image.

To facilitate the reproducibility of the presented results, the source code of the proposed method and test environments written in MATLAB R2020a environment are available at: https://github.com/Skythianos/GSF-IQA, accessed on 5 February 2021. 

## Figures and Tables

**Table 1:** Summary of features applied in the introduced no-reference image quality assessment (NR-IQA) algorithm. Quality-aware features proposed in this study are typed in bold.

Feature Number	Input	Feature	Length of Feature
**f1–f16**	Local fractal dimension map	normalized histogram, skewness, kurtosis, entropy, median, spread, std	16
**f17–f32**	Horizontal wavelet coefficients	normalized FDD, symmetric KL, skewness, kurtosis, entropy, median, spread, std	16
**f33–f48**	Vertical wavelet coefficients	normalized FDD, symmetric KL, skewness, kurtosis, entropy, median, spread, std	16
**f49–f64**	Diagonal wavelet coefficients	normalized FDD, symmetric KL, skewness, kurtosis, entropy, median, spread, std	16
**f65–f80**	DCT coefficients	normalized FDD, symmetric KL, skewness, kurtosis, entropy, median, spread, std	16
**f81–f96**	Singular values	normalized FDD, symmetric KL, skewness, kurtosis, entropy, median, spread, std	16
**f97–f103**	Bilaplacian maps of *Y* channel	histogram variance	7
**f104–f110**	Bilaplacian maps of Cb channel	histogram variance	7
**f111–f117**	Bilaplacian maps of Cr channel	histogram variance	7
**f118–f125**	Sobel edge map	image moments	8
f126	RO map [36]	histogram variance	1
f127	RM map [36]	histogram variance	1
f128	GM map [36]	histogram variance	1
f129	RGB image	colorfulness [37]	1
f130	Grayscale image	sharpness [38]	1
f131	RGB image	dark channel feature [39]	1
f132	RGB image	contrast [40]	1

**Table 2:** Average first digit distribution (FDD) of singular values in the KADID-10k [46] database with respect to the five different distortion levels of KADID-10k. Level 1 stands for the lowest level of distortion, while Level 5 denotes the highest distortion. The column sKL indicates the symmetric Kullback–Leibler divergence between the actual FDD and the prediction of Benford’s law.

	1	2	3	4	5	6	7	8	9	*sKL*
Level 1	0.307	0.184	0.126	0.095	0.076	0.064	0.055	0.049	0.044	8.52 × 10^−4^
Level 2	0.306	0.181	0.124	0.095	0.077	0.065	0.057	0.050	0.045	3.08 × 10^−4^
Level 3	0.312	0.182	0.123	0.092	0.075	0.064	0.056	0.050	0.046	8.59 × 10^−4^
Level 4	0.317	0.185	0.124	0.090	0.072	0.062	0.055	0.049	0.045	0.002
Level 5	0.315	0.192	0.128	0.092	0.071	0.060	0.053	0.048	0.044	0.004
Benford distribution	0.301	0.176	0.125	0.097	0.079	0.067	0.058	0.051	0.046	0

**Table 3:** The average values of perceptual features in the KADID-10k [46] database with respect to the five different distortion levels. Level 1 stands for the lowest level of distortion, while Level 5 denotes the highest distortion.

	CF	Sharpness	DCF	GCF
Reference	0.2430	0.1635	0.1655	7.3100
Level 1	0.2423	0.1640	0.1678	7.4314
Level 2	0.2590	0.1580	0.1588	7.5798
Level 3	0.2663	0.1526	0.1581	7.5652
Level 4	0.2732	0.1497	0.1573	7.6172
Level 5	0.2857	0.1458	0.1534	7.6995

**Table 4:** Publicly available IQA benchmark databases used in this paper.

Database	#Distorted Images	Distortion Type	#Distortion Types	#Ref. Images	Resolution
CSIQ [58]	866	artificial	4-5	30	512×512
MDID [59]	1600	artificial	5	20	512×384
KADID-10k [46]	10,125	artificial	25	81	512×384
LIVE In the Wild [35]	1162	authentic	-	-	500×500
KonIQ-10k [60]	10,073	authentic	-	-	1024×768

**Table 5:** Comparison of *GSF-IQA* to the state-of-the-art on artificial distortions. Mean PLCC and SROCC are measured over 100 random train–test splits with respect to the reference images. Best results are typed in bold, second best results are typed in italic.

	CSIQ [58]		MDID [59]		KADID-10K [46]	
Method	PLCC	SROCC	PLCC	SROCC	PLCC	SROCC
BLIINDS-II [66]	0.763	0.718	0.676	0.677	0.548	0.530
BMPRI [67]	0.785	0.737	0.757	0.751	0.554	0.530
BRISQUE [6]	0.613	0.531	0.612	0.618	0.383	0.386
CurveletQA [9]	0.738	0.707	0.671	0.673	0.473	0.450
DIIVINE [68]	0.654	0.635	0.713	0.722	0.423	0.428
ENIQA [69]	0.838	0.807	0.747	0.751	0.634	0.636
GRAD-LOG-CP [70]	0.786	0.766	0.608	0.628	0.585	0.566
NBIQA [71]	0.831	0.794	0.760	*0.768*	0.635	0.626
PIQE [4]	0.644	0.522	0.269	0.253	0.289	0.237
OG-IQA [36]	0.749	0.696	0.729	0.714	0.477	0.440
SPF-IQA [16]	*0.860*	*0.830*	0.727	0.725	*0.717*	*0.708*
SSEQ [72]	0.710	0.642	*0.763*	0.762	0.453	0.433
*GSF-IQA*	**0.875**	**0.840**	**0.781**	**0.773**	**0.737**	**0.725**

**Table 6:** Comparison of *GSF-IQA* to the state-of-the-art on authentic distortions. Mean PLCC and SROCC are measured over 100 random train–test splits. Best results are typed in bold, second best results are typed in italic.

	LIVE In the Wild [35]		KonIQ-10k [60]	
Method	PLCC	SROCC	PLCC	SROCC
BLIINDS-II [66]	0.450	0.419	0.571	0.575
BMPRI [67]	0.521	0.480	0.636	0.619
BRISQUE [6]	0.503	0.487	0.702	0.676
CurveletQA [9]	**0.620**	**0.611**	0.728	0.716
DIIVINE [68]	0.602	0.579	0.709	0.692
ENIQA [69]	0.578	0.554	0.758	0.744
GRAD-LOG-CP [70]	0.579	0.557	0.705	0.698
NBIQA [71]	0.607	0.593	*0.770*	*0.748*
PIQE [4]	0.171	0.108	0.206	0.245
OG-IQA [36]	0.526	0.497	0.652	0.635
SPF-IQA [16]	0.592	0.563	0.759	0.740
SSEQ [72]	0.469	0.429	0.584	0.573
*GSF-IQA*	*0.618*	*0.595*	**0.784**	**0.752**

**Table 7:** One-sided *t*-test. Symbol ‘1’ means that the proposed *GSF-IQA* method is statistically better than the NR-IQA method in the row on the IQA benchmark database in the column. Symbol ‘-’ is used when there is no significant difference.

	CSIQ [58]	MDID [59]	KADID-10K [46]	LIVE In the Wild [35]	KonIQ-10k [60]
BLIINDS-II [66]	1	1	1	1	1
BMPRI [67]	1	1	1	1	1
BRISQUE [6]	1	1	1	1	1
CurveletQA [9]	1	1	1	-	1
DIIVINE [68]	1	1	1	1	1
ENIQA [69]	1	1	1	1	1
GRAD-LOG-CP [70]	1	1	1	1	1
NBIQA [71]	1	1	1	1	-
PIQE [4]	1	1	1	1	1
OG-IQA [36]	1	1	1	1	1
SPF-IQA [16]	1	1	1	1	1
SSEQ [72]	1	1	1	1	1

**Table 8:** Comparison of *GSF-IQA* to the state-of-the-art. Weighted and direct average of measured PLCC and SROCC values are reported. Best results are typed in bold, second best results are typed in italic.

	Weighted Average		Direct Average	
Method	PLCC	SROCC	PLCC	SROCC
BLIINDS-II [66]	0.569	0.560	0.602	0.584
BMPRI [67]	0.609	0.588	0.651	0.623
BRISQUE [6]	0.547	0.534	0.563	0.540
CurveletQA [9]	0.611	0.595	0.646	0.631
DIIVINE [68]	0.581	0.574	0.620	0.611
ENIQA [69]	0.699	0.692	0.711	0.698
GRAD-LOG-CP [70]	0.644	0.633	0.653	0.643
NBIQA [71]	0.706	0.692	0.721	0.706
PIQE [4]	0.260	0.246	0.316	0.273
OG-IQA [36]	0.580	0.553	0.627	0.596
SPF-IQA [16]	*0.735*	*0.720*	*0.731*	*0.713*
SSEQ [72]	0.539	0.522	0.596	0.568
*GSF-IQA*	**0.759**	**0.737**	**0.759**	**0.737**

**Table 9:** Mean SROCC value comparison on different distortion types of the KADID-10k [46] database. Measured over 100 random train–test splits with respect to the reference images. The best results are typed in bold.

Dist. Type	BLIINDS-II [66]	BMPRI [67]	CurveletQA [9]	ENIQA [69]	GRAD-LOG-CP [70]	NBIQA [71]	OG-IQA [36]	SPF-IQA [16]	SSEQ [72]	GSF-IQA
GB	0.789	0.839	0.806	0.785	0.809	0.843	0.841	0.835	0.714	**0.873**
LB	0.755	0.815	**0.850**	0.797	0.808	0.845	0.804	0.802	0.739	0.800
MB	0.416	0.390	0.720	0.574	0.513	**0.749**	0.340	0.545	0.368	0.640
CD	0.519	0.445	0.270	0.691	0.416	0.633	0.289	**0.791**	0.422	0.750
CS	0.023	0.106	0.113	0.163	0.066	0.001	0.112	0.324	0.050	**0.348**
CQ	0.476	0.667	0.628	0.644	0.677	0.690	0.534	0.720	0.551	**0.759**
CSA1	0.126	0.099	0.040	0.064	0.007	0.024	0.046	0.086	0.108	**0.161**
CSA2	0.509	0.439	0.038	0.675	0.333	0.641	0.175	**0.759**	0.213	0.695
JP2K	0.636	0.616	0.605	0.634	0.670	0.694	0.566	0.605	0.455	**0.699**
JPEG	0.759	**0.817**	0.615	0.773	0.783	0.803	0.742	0.806	0.689	0.795
WN	0.544	0.841	0.723	0.769	0.846	0.767	0.723	**0.890**	0.638	0.883
WNCC	0.683	0.769	0.756	0.796	0.861	0.789	0.684	**0.912**	0.674	0.900
IN	0.609	0.457	0.609	0.618	0.710	0.649	0.557	0.701	0.581	**0.798**
MN	0.589	0.606	0.624	0.722	0.722	0.745	0.673	0.773	0.602	**0.829**
Denoise	0.687	0.814	0.772	0.809	0.826	0.864	0.712	**0.882**	0.617	0.835
Brighten	0.397	0.437	0.403	0.515	0.449	0.489	0.216	**0.643**	0.277	0.624
Darken	0.425	0.372	0.198	0.361	0.367	**0.476**	0.264	0.386	0.312	0.307
MS	0.214	0.206	0.055	0.112	0.138	**0.268**	0.094	0.139	0.099	0.149
Jitter	0.820	0.701	0.594	0.645	0.790	0.777	0.483	0.715	0.539	**0.821**
NEP	0.042	−0.042	0.038	0.019	0.076	0.016	0.077	0.076	−0.002	**0.172**
Pixelate	0.576	0.526	0.113	0.472	0.681	0.567	0.280	0.716	0.460	**0.735**
Quantization	0.304	0.304	0.350	0.548	0.578	0.464	0.531	**0.688**	0.202	0.596
CB	0.176	0.151	0.072	0.126	0.306	0.158	0.076	**0.377**	0.167	0.291
HS	0.620	0.544	0.622	0.709	0.701	0.650	0.585	0.819	0.586	**0.849**
CC	0.116	0.129	−0.002	0.188	0.136	0.230	0.172	0.226	0.061	**0.273**
All	0.530	0.530	0.450	0.636	0.566	0.626	0.440	0.708	0.433	**0.725**

**Table 10:** Mean SROCC value comparison on different distortion levels of the KADID-10k [46] database. Measured over 100 random train–test splits with respect to the reference images. The best results are typed in bold.

Dist. Type	BLIINDS-II [66]	BMPRI [67]	CurveletQA [9]	ENIQA [69]	GRAD-LOG-CP [70]	NBIQA [71]	OG-IQA [36]	SPF-IQA [16]	SSEQ [72]	GSF-IQA
Level 1	0.172	0.093	0.082	0.127	0.103	0.133	0.087	0.212	0.007	**0.217**
Level 2	0.228	0.259	0.186	0.373	0.298	0.366	0.223	0.458	0.127	**0.490**
Level 3	0.358	0.383	0.309	0.505	0.403	0.445	0.282	0.603	0.246	**0.642**
Level 4	0.535	0.488	0.417	0.610	0.513	0.595	0.374	0.691	0.363	**0.694**
Level 5	0.629	0.584	0.532	0.688	0.605	0.678	0.494	0.741	0.548	**0.747**
All	0.530	0.530	0.450	0.636	0.566	0.626	0.440	0.708	0.433	**0.725**

## Data Availability

No new data were created or analysed in this study. Data sharing is not applicable to this article.
